# Risk Stratification by Combination of Heart and Lung Dose in Locally Advanced Non-Small-Cell Lung Cancer after Radiotherapy

**DOI:** 10.3390/cancers16193255

**Published:** 2024-09-24

**Authors:** Yui Watanabe, Yutaro Koide, Hidetoshi Shimizu, Takahiro Aoyama, Yurika Shindo, Shingo Hashimoto, Hiroyuki Tachibana, Takeshi Kodaira

**Affiliations:** 1 Department of Radiation Oncology, Aichi Cancer Center Hospital, Nagoya 464-8681, Aichi, Japan; ykoide@aichi-cc.jp (Y.K.); hishimizu@aichi-cc.jp (H.S.); aoyamat@aichi-cc.jp (T.A.); y.shindo@aichi-cc.jp (Y.S.); s.hashimoto@aichi-cc.jp (S.H.); tchbn@aichi-cc.jp (H.T.); 109103@aichi-cc.jp (T.K.); 2 Department of Radiation Oncology, Daiyukai General Hospital, Ichinomiya 491-8551, Aichi, Japan

**Keywords:** non-small-cell lung cancer, radiotherapy, survival rates, prognostic factors

## Abstract

**Simple Summary:**

This study investigated the impact of radiation doses to the heart and lungs on overall survival (OS) in patients with locally advanced non-small-cell lung cancer (LA-NSCLC) treated with radiotherapy. An analysis of 120 patients, each with exactly three years of follow-up, revealed that higher radiation doses to the heart and lungs were significantly associated with worse OS. Importantly, the combination of heart and lung radiation doses provided enhanced and more detailed risk stratification, making it a more powerful predictor of poor survival than individual doses alone. These findings suggest that minimizing combined radiation exposure to both organs may improve survival outcomes in LA-NSCLC patients.

**Abstract:**

**Background/Objectives:** Despite advancements in treatment for patients with unresectable locally advanced non-small cell lung cancer (LA-NSCLC), overall survival (OS) remains poor. The specific effects of varying heart and lung doses on OS in LA-NSCLC patients have not been thoroughly investigated, especially their combined impact on survival. This study aimed to examine the impact on OS of both individual and combined heart and lung doses in patients with LA-NSCLC treated with radiotherapy over a three-year follow-up period. **Methods:** A total of 120 patients who received definitive radiotherapy for LA-NSCLC (stage III, 92.5%) from January 2015 to January 2020 were retrospectively reviewed. The endpoint in this study was OS. Each patient was followed for a fixed period of three years. **Results:** Univariate Cox regression analysis showed that OS was significantly related to mean heart dose (MHD, hazard ratio [HR], 3.4 [1.8–6.3]; *p* < 0.001), pericardium V40 (HR, 3.2 [1.7–6.0]; *p* < 0.001), and total lung V20 (HR, 2.6 [1.4–5.0]; *p* = 0.003), and these were independent predictors for worse OS in multivariate analysis. Kaplan–Meier curve analysis with log-rank tests revealed that survival was significantly worse in patients with higher MHD (*p* < 0.001), pericardium V40 (*p* < 0.001), and total lung V20 (*p* = 0.002). Combining MHD and total lung V20, and pericardium V40 and total lung V20 provided enhanced risk stratification for OS (*p* < 0.001 for both combinations). **Conclusions:** The combination of heart and lung doses provided enhanced and more detailed risk stratification in prediction of OS for a fixed period of three years in LA-NSCLC patients treated with radiotherapy.

## 1. Introduction

Inoperable locally advanced non-small-cell lung cancer (LA-NSCLC) is typically addressed with a multimodal treatment approach. Definitive radiotherapy, often combined with chemotherapy and immunotherapy, forms the cornerstone in this strategy [1,2]. Radiation therapy (RT) aims to deliver a high radiation dose to the tumor while minimizing exposure to surrounding healthy tissue. However, due to the proximity of the tumor to critical organs such as the heart and lungs, some level of radiation exposure to these organs at risk (OARs) is inevitable. Radiation-induced injury to the lung is a well-documented concern, with a substantial body of research dedicated to understanding and reducing its effects [3,4,5].

The impact of radiation on the heart has also gained attention, supported by an increasing body of evidence linking RT for breast cancer or lymphoma to long-term cardiac-related mortality [6,7]. This has led to an increased interest in cardiac toxicity among patients receiving RT for lung cancer. A landmark trial in this area, the Radiation Therapy Oncology Group (RTOG) 0617 trial [3], investigated the effects of high-dose versus standard-dose thoracic radiation in patients with NSCLC. Surprisingly, this study revealed worse overall survival (OS) in the high-dose arm, spurring numerous secondary analyses to investigate the potential causes, with a focus on radiation dose to the heart and other OARs [8,9,10,11,12,13,14,15,16]. Follow-up studies have sought to verify the findings from RTOG0617 in independent cohorts but have shown mixed results. Some studies supported the RTOG 0617 findings, showing that higher radiation doses to the heart correlate with poorer outcomes, while others failed to demonstrate a clear relationship [17,18,19,20]. The ongoing debate implies the significance of cardiac radiation dose in the prognosis of NSCLC patients.

The variability in lung and heart radiation doses is influenced by several factors, including tumor size, location, and the chosen radiation therapy technique. Intensity-modulated radiation therapy (IMRT), an advanced radiotherapy technique, has become increasingly prevalent due to its precision in targeting tumors. IMRT has the potential to significantly reduce radiation exposure to the heart and lung compared to traditional three-dimensional conformal radiation therapy (3D-CRT) [9], thereby potentially diminishing the risk of organ toxicity. A recent study [21] has implied the importance of incorporating both heart and lung dose parameters into prognostic models. These findings suggested that minimizing the combined radiation exposure to the heart and lungs could significantly improve survival outcomes in patients with LA-NSCLC. However, despite the growing recognition of this issue, the combined effects of heart and lung radiation doses on OS have not been fully explored in the current literature. Examining both the individual and combined effects of lung and heart irradiation is essential for refining radiotherapy protocols and developing personalized treatment plans that balance tumor control with organ preservation. This study aimed to assess how both individual and combined lung and heart doses affect survival in NSCLC patients treated with RT.

## 2. Materials and Methods

### 2.1. Study Design

Patients who underwent definitive radiotherapy for NSCLC, with or without chemotherapy, between January 2015 and January 2020, were retrospectively analyzed in this study. The clinical records and dosimetry data from these patients were examined at a single academic institution. This study was conducted according to an institutional review board-approved protocol (approval number: 2024-0-070). All patient data were anonymized, and analyses followed the applicable institutional guidelines and regulations.

Using our institution’s radiation therapy database, we identified NSCLC patients treated with definitive radiotherapy between January 2015 and January 2020. Patients were included if they had stage II to III NSCLC and no distant metastasis before treatment. A total of 231 patients met the inclusion criteria. Of these, we excluded 111 patients who met the following criteria: (1) preoperative or postoperative RT (n = 53); (2) neoadjuvant chemotherapy (n = 3); (3) fractionated radiotherapy with doses other than 2 Gy per fraction, such as 3 Gy (n = 8); (4) canceled radiotherapy due to toxicity or general condition (n = 5); and (5) follow-up loss within three years (n = 42). Therefore, the study population comprised 120 patients.

The contours of patients’ OARs (the heart, pericardium, left ventricle, and right and left lungs) were reviewed and recontoured if necessary. Specifically, for the heart, they were reviewed and recontoured as needed using the guidelines set out by Wheatley et al., specifically developed for the secondary analysis of RTOG 0617 [22].

### 2.2. Treatments

All patients were treated with definitive, fractionated radiotherapy, either with or without concurrent chemotherapy. Computed tomography (CT) simulation images with a slice thickness of 2 or 3 mm were acquired while the patient was in the supine position using the Aquilion LB CT system (Canon Medical Systems, Ohtawara, Tochigi, Japan). Treatment planning was carried out based on CT simulation scans of the thorax. Experienced radiation therapists outlined the target volumes and OARs, including automatic thresholding for lung delineation, while the heart was delineated as previously defined.

Treatment planning systems used for developing the radiotherapy plans included Pinnacle (version 9.10; Philips Medical Systems, Fitchburg, WI, USA), RayStation (version 10.0; RaySearch Laboratories, Stockholm, Sweden), and Xio (version 5.10; Elekta, Stockholm, Sweden). The total radiation dose ranged from 56 to 66 Gy, delivered over 28 to 33 fractions of 2 Gy per fraction, 5 days a week. Radiotherapy was administered using IMRT or 3D-CRT techniques, with linear accelerators delivering 6- or 10-megavolt photons. The equipment utilized for radiation delivery included TrueBeam (Varian Medical Systems, Palo Alto, CA, USA), Synergy (Elekta, Stockholm, Sweden), and Clinac 21EX (Varian Medical Systems, Palo Alto, CA, USA).

Dose–volume histogram (DVH) data were collected from the treatment plans, and the relevant dosimetric parameters were calculated. OAR doses were quantified using the dosimetric index VD, defined as the percentage of the organ’s volume receiving a dose ≥ D in Gy, such as V50, V40, and V30.

Chemotherapy typically consisted of platinum-based chemotherapy delivered concurrently with radiation therapy. Most patients (n = 113, 94.2%) received chemotherapy. Among those patients, 105 received platinum-based doublet regimens, including cisplatin or carboplatin plus a cytotoxic agent (paclitaxel, pemetrexed, docetaxel, S-1, vinorelbine, or etoposide). Only 5 and 3 patients received single-agent carboplatin and gefitinib, respectively.

### 2.3. Statistical Analysis

Follow-up time was calculated from the start of RT. The endpoint for this study was OS, defined as follows: (1) the time from the start of RT to death from any cause if the patient died within three years after RT or (2) three years if the patient was alive three years after RT. The receiver operating characteristic (ROC) curve analysis was performed to investigate the optimal cutoff values of heart and lung dose parameters, which were identified as the thresholds that maximize the Youden index. OS rates were calculated using the Kaplan–Meier method, and differences between groups were evaluated with the log-rank test. To estimate hazard ratios (HRs) along with their 95% confidence intervals, Cox proportional hazards regression models were applied for both univariate and multivariate analyses. In the multivariate analysis, three models were constructed: each model included a significant variable with the highest HR among the heart dose parameters (heart, pericardium, and LV) and one with the highest HR among the lung dose parameters in the univariate analysis. A *p*-value of 0.05 or less was considered statistically significant. All analyses were performed using R software version 3.6.1 (The R Foundation for Statistical Computing, Vienna, Austria).

## 3. Results

### 3.1. Patient Characteristics and Radiation Dose Parameters for Heart and Lungs

Table 1 summarizes the characteristics of the 120 patients analyzed in this study. The median age was 68 years (range, 32–84 years), with 61.7% of patients aged 65 years or older. The study population was predominantly male (76.7%) and had a good performance status, with 98.3% having an Eastern Cooperative Oncology Group score of 0 or 1. Most patients were diagnosed with stage III (92.5%), and a large majority (94.2%) received chemotherapy. Adenocarcinoma was the most common histological subtype (51.7%), followed by squamous cell carcinoma (33.3%). Nearly all patients (97.5%) underwent radiation therapy and, additionally, 87.5% of patients were treated with 3D-CRT. Table 2 presents the radiation dose parameters for the heart and lungs in the analyzed patients.

### 3.2. Univariate and Multivariable Models for Overall Survival

Table 3 presents the results of a univariate Cox proportional hazards regression analysis assessing various factors influencing patient outcomes. Significant predictors of worse survival included age ≥ 65 years (HR, 2.1), male (HR, 3.3), and higher TNM stage (HR, 2.0). Concurrent chemotherapy and the use of Durvalumab after chemoradiotherapy were associated with improved survival (HR, 0.3 for both). Several radiation dose parameters were also significant, with higher heart doses, including a heart mean dose ≥ 1188 cGy (HR, 3.4), pericardium V40 ≥ 24.5% (HR, 3.2), and a left ventricular (LV) mean dose ≥ 155 cGy (HR, 2.3), showing strong associations with poorer outcomes. Similarly, elevated lung doses, such as a total lung V20 ≥ 25.38% (HR, 2.6), were linked to worse survival. The cutoff values for the dose parameters were determined through ROC curve analysis to optimize the prediction for overall survival.

Table 4 presents the results of the multivariate Cox proportional hazards regression analysis, highlighting the significant predictors of patient survival across three different models. In Model 1, both the mean heart dose (MHD) and the total lung V20 were identified as significant predictors of poorer outcomes, with HRs of 2.8 and 2.0, respectively. Similarly, in Model 2, the pericardium V40 and the total lung V20 remained significant, with HRs of 2.6 and 2.0, respectively. In Model 3, while the total lung V20 continued to show a significant impact on survival (HR, 2.1), the left ventricular mean dose did not reach statistical significance (HR, 1.7).

### 3.3. Kaplan–Meier Curve Analysis for Each Lung and Heart Dose and Their Combination

We found that the three-year survival rates of each clinical stage (the 8th Edition of the Union for International Cancer Control TNM classification) in this study population were 89% (8/9), 82% (28/34), 58% (38/65), and 50% (6/12) at stages IIB, IIIA, IIIB, and IIIC, respectively.

**Figure 1:**
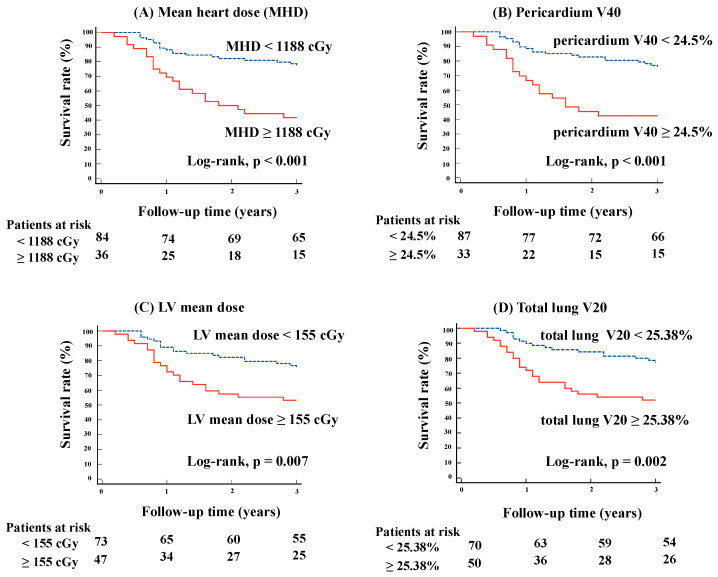
Kaplan–Meier curve analysis by heart and lung dose parameters. The figures present a Kaplan–Meier curve analysis of survival rates, stratified by heart and lung dose parameters. The analysis includes (**A**) MHD, (**B**) pericardium V40, (**C**) LV mean dose, and (**D**) total lung V20. For MHD, the survival rate was significantly lower in patients receiving doses ≥ 1188 cGy compared to those receiving < 1188 cGy (*p* < 0.001). Similarly, higher doses to the pericardium (V40 ≥ 24.5%), LV (mean dose ≥ 155 cGy), and total lung (V20 ≥ 25.38%) were associated with reduced survival, with *p*-values of < 0.001, 0.007, and 0.002, respectively. MHD, mean heart dose; LV, left ventricular; Vx, volume of the organ receiving at least xGy.

Based on the results of the univariate analysis described above, we included MHD, pericardium V40, LV mean dose, and total lung V20 as parameters for the Kaplan–Meier analysis, using cutoff values of 1188 cGy, 24.5%, 155 cGy, and 25.38%, respectively. We generated and analyzed Kaplan–Meier curves for OS using these cutoff values (Figure 1). In Figure 1, patients with higher values for MHD, pericardium V40, LV mean dose, and total lung V20 had significantly worse survival outcomes compared to those with lower values (*p* < 0.001, *p* < 0.001, *p* = 0.007, and *p* = 0.002, respectively).

**Figure 2:**
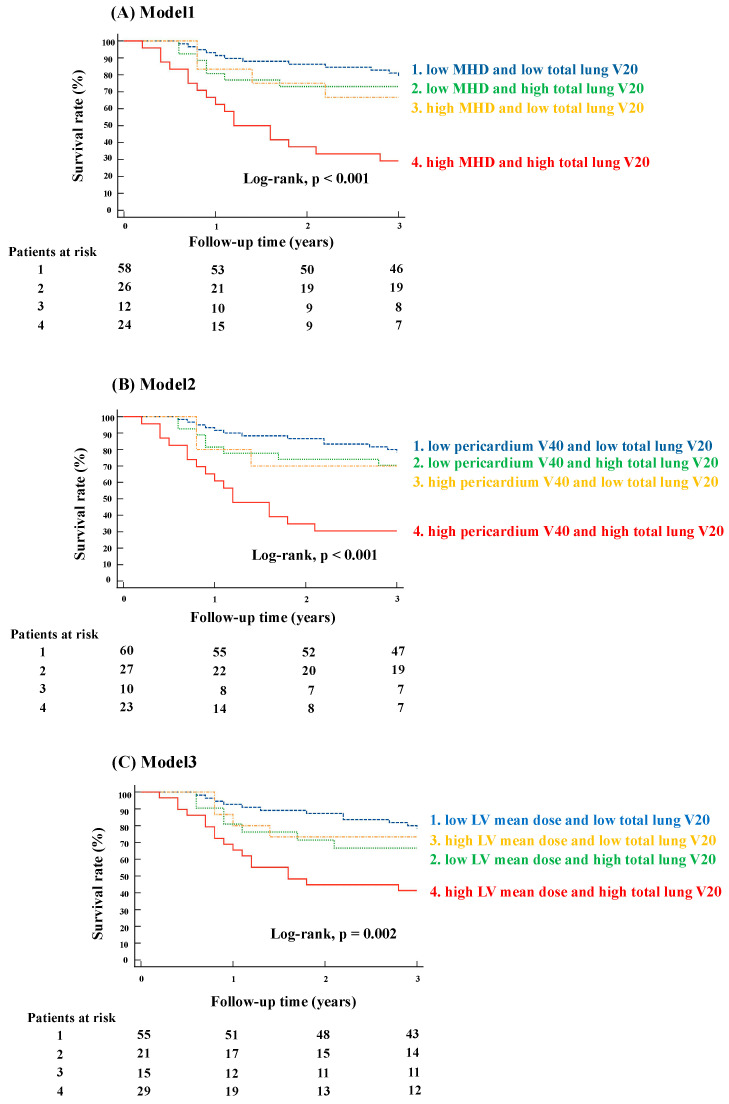
Kaplan–Meier curve by combination of heart and dose parameters. The figures show a Kaplan–Meier curve analysis of survival rates based on combinations of heart and lung dose parameters, specifically focusing on MHD, pericardium V40, and LV mean dose, in conjunction with total lung V20. (**A**) (Model 1) focuses on the relationship between MHD and total lung V20 in determining survival rates. The analysis divided the patients into four groups based on their MHD and total lung V20 values: (1) low MHD and low total lung V20, (2) low MHD and high total lung V20, (3) high MHD and low total lung V20, and (4) high MHD and high total lung V20. The significant difference in survival outcomes across these groups was shown by a log-rank test with a *p*-value of <0.001. (**B**) (Model 2) examines the impact of pericardium V40 and total lung V20 on survival rates. The patients were divided into four groups based on whether they had low or high pericardium V40 in combination with low or high total lung V20: (1) low pericardium V40 and low total lung V20, (2) low pericardium V40 and high total lung V20, (3) high pericardium V40 and low total lung V20, and (4) high pericardium V40 and high total lung V20. The significant difference in survival rates across these groups is highlighted by a log-rank test with a *p*-value of <0.001. (**C**) (Model 3) analyzes the relationship between LV mean dose and total lung V20 on survival rates. The patients were categorized into four groups based on their LV mean dose and total lung V20 levels: (1) low LV mean dose and low total lung V20, (2) low LV mean dose and high total lung V20, (3) high LV mean dose and low total lung V20, and (4) high LV mean dose and high total lung V20. The log-rank test revealed a significant difference in survival between these groups with a *p*-value of 0.002. OS, overall survival; MHD, mean heart dose; LV, left ventricular; Vx, volume of the organ receiving at least xGy.

Figure 2 illustrates OS stratification based on combinations of heart and lung dose parameters. The patients were divided into four groups using two parameters with their respective cutoff values: one heart-related and the other lung-related. Group 1 had low doses for both parameters, Group 2 had low heart-related and high lung-related doses, Group 3 had high heart-related and low lung-related doses, and Group 4 had high doses for both parameters. As shown in Figure 2, the Kaplan–Meier curve analysis demonstrated that the patients who received higher doses to both the heart and lungs had worse OS compared to those with lower doses for both parameters. Additionally, the patients with high doses for either parameter had OS trends that fell between the extremes of the two groups. The analyses in Figure 2 show that the combination of radiation doses for (A) MHD and total lung V20, (B) pericardium V40 and total lung V20, and (C) LV mean dose and total lung V20 provided enhanced and more detailed risk stratification for OS (*p* < 0.001, *p* < 0.001, and *p* = 0.002, respectively).

## 4. Discussion

This research assessed how radiation doses to the lungs and heart impacted survival outcomes in NSCLC patients treated with RT. The principal conclusions were as follows: (1) elevated radiation doses to the lungs and heart were associated with poorer OS in patients with NSCLC; (2) the multivariate analysis confirmed that radiation doses to the lungs and heart were both independent predictors of adverse OS; and (3) the combination of radiation doses for the lungs and heart provided enhanced and more detailed risk stratification for OS, showing that patients with higher doses for both the lung and heart had the worst OS.

Lung cancer continues to be the most frequent cause of cancer-related mortality globally [23]. According to the guidelines set forth by the National Comprehensive Cancer Network (NCCN) [1], the recommended treatment for locally advanced and inoperable NSCLC involves the concurrent use of platinum-based chemotherapy and thoracic external beam radiation. The standard prescribed radiation dose falls within the range of 60 to 70 Gy, administered in daily fractions of 2 Gy. Numerous phase III randomized clinical trials have demonstrated that concurrent chemoradiotherapy (CCRT) provides a clear survival advantage over non-CRT such as sequential chemotherapy or radiotherapy alone for patients with LA-NSCLC [24,25]. Furthermore, consolidation therapy with durvalumab after CCRT has shown a significant improvement in OS for patients with unresectable LA-NSCLC [2]. Despite these therapeutic advancements, the overall survival rate of LA-NSCLC patients remains suboptimal, indicating the necessity for identifying and incorporating additional prognostic factors into treatment strategies.

The RTOG 0617 trial [3], a large-scale, multi-center, randomized phase III study, assessed the impact of dose-escalated radiotherapy (74 Gy versus 60 Gy) combined with concurrent chemotherapy. This study revealed that patients receiving the higher radiation dose had a median OS of 20.3 months, which was shorter than the 28.7 months observed in the standard-dose group. This outcome raised significant concerns due to the higher incidence of treatment-related deaths in the high-dose group, particularly from toxicity affecting OARs, notably the heart. Subsequent research has produced inconsistent findings regarding the influence of heart dose on OS, with some studies identifying it as a negative prognostic factor, while others found no such correlation [8,9,10,11,12,13,14,15,16]. Our study confirmed that increased radiation doses to the heart were linked to reduced OS in NSCLC patients undergoing radiotherapy, with the multivariate analyses identifying heart dose as an independent factor associated with worse outcomes. Further secondary analyses of RTOG 0617, as well as other studies such as those conducted by Speirs et al. [9], emphasized the importance of heart dose in patient prognosis. In addition, a meta-analysis of ten studies, including both randomized controlled trials and cohort studies, found that heart volumes receiving doses of ≥5 Gy (HV5), ≥30 Gy (HV30), and ≥50 Gy (HV50), along with mean heart dose, were all significantly associated with decreased OS and increased risk of cardiac complications [26]. This implies the critical need to limit radiation exposure to the heart during thoracic RT to improve survival outcomes for thoracic cancer patients.

Our findings also indicated that higher radiation doses to the lung were associated with poorer survival in NSCLC patients receiving radiotherapy. The univariate analysis showed that metrics such as total lung V20, V5, and ipsilateral mean lung dose were correlated with OS. The multivariate analysis further demonstrated that lung dose served as an independent predictor of unfavorable OS. Previous studies have primarily focused on the effects of lung dose on radiation pneumonitis (RP) or fibrosis, with limited emphasis on survival as an endpoint [5,27]. For example, the RTOG 0617 study did not observe a significant relationship between lung V5 and OS, and there is a relative paucity of research directly examining the impact of lung dose on survival outcomes. In a retrospective review, Shen et al. [11] studied 130 stage III NSCLC patients treated with volumetric modulated arc therapy (VMAT) and found that both RP and heart V15 were predictive of worse survival in these patients. Similarly, Heo et al. [28] analyzed 178 NSCLC patients undergoing postoperative radiotherapy, showing that elevated lung doses correlated with diminished survival rates. Xu et al. [29] examined the connection between radiation doses to the heart and lungs and OS in a cohort of 560 esophageal cancer patients who underwent CCRT, either with or without surgical intervention. Their findings demonstrated that both heart and lung doses were independent predictors of worse OS in these patients.

The specific effects of varying radiation doses to the lungs and heart and the combined impact on survival have not been fully explored. Our study found that not only do the individual doses to the lungs and heart affect survival outcomes but also that the combination of these doses provides a more refined risk stratification for OS. Patients receiving higher doses to both the lungs and heart showed the poorest survival. With the increasing use of IMRT, a more advanced radiation technique, it has become easier to reduce doses to OARs, compared to traditional 3D-CRT. While reducing doses to both the lungs and heart offers the most benefit, in some cases lowering either one without compromising treatment efficacy is still advantageous. The multivariate analysis in our study revealed that higher radiation doses to both the heart and lungs independently predicted poorer OS. The cutoff values identified—1188 cGy for MHD, 1192.6 cGy for mean lung dose, and 25.38% for total lung V20—were lower than the commonly used dose limits [1,30,31] in clinical treatment plans. This suggests that further dose reductions to both the heart and lungs, while maintaining adequate tumor targeting, could improve survival even when typical constraints are met.

There are several limitations to this study. First, as a retrospective, single-center study, the generalizability of the findings is limited. Second, we included patients who received varying chemotherapy regimens and a few who were treated with radiotherapy alone, leading to some inconsistency in treatment approaches. Third, we did not investigate heart and lung events, or damage or impacts to heart substructures other than the left ventricle. Further research is needed to explore how these additional factors influence survival outcomes. Fourth, this study did not include lower-stage patients who would typically be eligible for stereotactic body radiation therapy, which is well known for its precision in delivering high doses of radiation to tumors in fewer fractions. Due to the high dose per fraction, even small amounts of radiation leakage to nearby OARs can result in significant exposure, and therefore, careful dose planning and stricter dose constraints may be essential, particularly for tumors near the heart. Fifth, in this study, we prioritized the analysis of radiation dose parameters and their impact on OS and did not extensively investigate the influence of non-radiation-related patient factors, such as age, NSCLC subtype, or chemotherapy dosage, on survival outcomes. Further studies are warranted to explore these factors more comprehensively, including both radiation dose parameters and broader patient characteristics, to provide a more detailed understanding of the elements influencing OS.

## 5. Conclusions

Irradiation doses for the heart and lungs were both independent predictors of worse OS in NSCLC patients treated with radiotherapy. Furthermore, the combination of radiation doses for the lung and heart provided enhanced and more detailed risk stratification for OS than the individual effects of lung and heart doses.

## Figures and Tables

**Table 1:** Patient characteristics.

Characteristics	No. of Patients (%) (n = 120)
Age	
<65	46 (38.3)
≥65	74 (61.7)
Sex	
Male	92 (76.7)
Female	28 (23.3)
ECOG performance status	
0	64 (53.3)
1	54 (45.0)
2	1 (0.8)
3	1 (0.8)
T stage *	
1	29 (24.2)
2	28 (23.3)
3	27 (22.5)
4	36 (30.0)
N stage *	
0	5 (4.2)
1	11 (9.2)
2	66 (55.0)
3	38 (31.7)
Clinical stage *	
IIB	9 (7.5)
IIIA	34 (28.3)
IIIB	65 (54.2)
IIIC	12 (10.0)
Histology	
Adenocarcinoma	62 (51.7)
Squamous cell	40 (33.3)
Other	18 (15.0)
Chemotherapy	
Yes	113 (94.2)
No	7 (5.8)
Durvalumab after CRT	
Yes	31 (25.8)
No	89 (74.2)
Radiation technique	
3D-CRT	105 (87.5)
IMRT	15 (12.5)
Prescribed dose/fractionation	
60 Gy/30 fr	111 (92.5)
64 Gy/32 fr	3 (2.5)
66 Gy/33 fr	3 (2.5)
58 Gy/29 fr	2 (1.7)
56 Gy/28 fr	1 (0.8)

IMRT, intensity-modulated radiation therapy; CRT, chemoradiotherapy; 3D-CRT, three-dimensional conformal radiation therapy. * TNM staging: the 8th Edition of the Union for International Cancer Control (UICC) TNM classification.

**Table 2:** Radiation dose parameters for the heart and lungs.

Dose Parameters	Median (Interquartile Range)
Heart Doses	
Heart mean dose (cGy)	741 (381–1500)
Heart V50 (%)	4.0 (0.1–12.9)
Heart V40 (%)	6.9 (1.0–16.6)
Heart V30 (%)	10.9 (3.0–20.5)
Pericardium mean dose (cGy)	1362 (1004–2021)
Pericardium V50 (%)	13.8 (7.0–20.4)
Pericardium V40 (%)	17.4 (11.0–26.2)
Pericardium V30 (%)	21.5 (16.0–31.0)
LV mean dose (cGy)	114 (53–284)
Lung Doses	
Total lung mean dose (cGy)	1277 (922–1527)
Total lung V20 (%)	23.2 (17.1–28.0)
Total lung V5 (%)	38.2 (29.3–44.0)
Ipsilateral lung mean dose (cGy)	2106 (1585–2557)
Ipsilateral lung V20 (%)	40.1 (30.9–48.0)
Ipsilateral lung V5 (%)	57.9 (48.2–68.0)
Contralateral lung mean dose (cGy)	384 (203–586)
Contralateral lung V20 (%)	5.5 (2.3–9.2)
Contralateral lung V5 (%)	16.6 (7.7–25.3)

LV, left ventricular; Vx, the percentage of the volume of the organ receiving at least xGy.

**Table 3:** Univariate Cox proportional regression analysis.

Variables	*p*-Value	HR (95% CI)
Patient Data		
Age ≥ 65	0.041	2.1 (1.0–4.3)
Male	0.024	3.3 (1.2–9.3)
ECOG PS (0, 1, 2, 3)	0.034	1.9 (1.0–3.4)
TNM stage (IIB, IIIA, IIIB, IIIC)	0.003	2.0 (1.3–3.1)
Adenocarcinoma (vs. SCC)	0.449	0.8 (0.4–1.5)
Concurrent chemotherapy	0.026	0.3 (0.1–0.9)
Durvalumab after CRT	0.024	0.3 (0.1–0.9)
Dose Data		
Heart Doses		
Heart mean dose (≥1188 cGy)	<0.001	3.4 (1.8–6.3)
Heart V50 (≥10.64%)	0.002	2.7 (1.4–5.0)
Heart V40 (≥14.08%)	0.001	2.8 (1.5–5.2)
Heart V30 (≥17.66%)	<0.001	3.1 (1.7–5.8)
Pericardium mean dose (≥1493.5 cGy)	0.002	2.8 (1.5–5.3)
Pericardium V50 (≥17%)	0.001	2.8 (1.5–5.3)
Pericardium V40 (≥24.5%)	<0.001	3.2 (1.7–6.0)
Pericardium V30 (≥23%)	0.001	2.9 (1.5–5.7)
LV mean dose (≥155 cGy)	0.009	2.3 (1.2–4.3)
Lung Doses		
Total lung mean dose (≥1192.6 cGy)	0.009	2.6 (1.3–5.4)
Total lung V20 (≥25.38%)	0.003	2.6 (1.4–5.0)
Total lung V5 (≥38.12%)	0.010	2.4 (1.2–4.6)
Ipsilateral lung mean dose (≥2191 cGy)	0.048	1.9 (1.0–3.5)
Ipsilateral lung V20 (≥43.89%)	0.021	2.1 (1.1–3.9)
Ipsilateral lung V5 (≥57%)	0.090	1.8 (0.9–3.4)
Contralateral lung mean dose (≥565.4 cGy)	0.165	1.6 (0.8–3.0)
Contralateral lung V20 (≥9%)	0.101	1.7 (0.9–3.2)
Contralateral lung V5 (≥11.7%)	0.141	0.6 (0.3–1.2)

SCC, squamous cell carcinoma; ECOG PS, Eastern Cooperative Oncology Group performance status; CRT, chemoradiotherapy; LV, left ventricular; Vx, the percentage of the volume of the organ receiving at least xGy; CI, confidence interval.

**Table 4:** Multivariate Cox proportional regression analysis.

	Model 1		Model 2		Model 3	
	*p*-Value	HR (95% CI)	*p*-Value	HR (95% CI)	*p*-Value	HR (95% CI)
MHD	0.002	2.8 (1.4–5.3)				
Pericardium V40			0.005	2.6 (1.3–5.0)		
LV mean dose					0.108	1.7 (0.9–3.4)
Total lung V20	0.042	2.0 (1.0–3.9)	0.041	2.0 (1.0–3.9)	0.028	2.1 (1.1–4.2)

MHD, mean heart dose; LV, left ventricular; Vx, the percentage of the volume of the organ receiving at least xGy; CI, confidence interval.

## Data Availability

The data presented in the present paper are available.

## Abbreviations

3D-CRT	three-dimensional conformal radiation therapy
CCRT	concurrent chemoradiotherapy
CRT	chemoradiotherapy
CT	computed tomography
DVH	dose–volume histogram
HR	hazard ratio
IMRT	intensity-modulated radiation therapy
LA-NSCLC	locally advanced non-small-cell lung cancer
LV	left ventricular
MHD	mean heart dose
NCCN	National Comprehensive Cancer Network
NSCLC	non-small-cell lung cancer
OARs	organs at risk
OS	overall survival
ROC	receiver operating characteristic
RP	radiation pneumonitis
RT	radiation therapy
RTOG	Radiation Therapy Oncology Group
Vx	the percentage of the volume of the organ receiving at least xGy
VMAT	volumetric modulated arc therapy
